# PLINs-mediated organelle interactions: a key of exercise-mediated improvement of skeletal muscle lipid metabolism disorders

**DOI:** 10.3389/fendo.2025.1700668

**Published:** 2025-11-03

**Authors:** Zhiyuan Liu, Yungang Zhao

**Affiliations:** Tianjin Key Laboratory of Exercise Physiology and Sports Medicine, Institute of Sport, Exercise and Health, Tianjin University of Sport, Tianjin, China

**Keywords:** perilipin, organelle interaction, skeletal muscle, exercise, lipid metabolism

## Abstract

Perilipins are essential structural proteins localized on the surface of lipid droplets, with perilipin 2, 3, and 5 exhibiting specific expression in skeletal muscle. Intramuscular lipids are predominantly stored within lipid droplets, tightly regulated by perilipins. Perilipin 3 primarily governs lipid droplet biogenesis, whereas Perilipin 2 and PLIN5 play critical roles in mediating lipolysis through lipid droplet-organelle interactions and in responding to exercise-induced signaling cascades. Acute exercise selectively depletes lipid droplets coated with Perilipin 2 and Perilipin 5 while inducing subcellular relocalization of perilipin proteins. In contrast, moderate-intensity continuous training and high-intensity interval training elicit adaptive alterations in skeletal muscle perilipins protein expression: Moderate-intensity continuous training significantly upregulates perilipin 2 and 5 expression, whereas high-intensity interval training specifically enhances perilipin5 expression, fostering enhanced physical interactions between lipid droplets and mitochondria, thereby mitigating ectopic lipid accumulation. This study elucidates the intricate regulatory mechanisms of perilipin-mediated organelle interactions under diverse exercise modalities and their contributions to optimizing skeletal muscle lipid metabolism, providing a robust theoretical framework for developing targeted exercise-based interventions and potential therapeutic targets for metabolic disorders.

## 1 Introduction

As a primary organ responsible for energy expenditure and movement execution, skeletal muscle holds a key position in maintaining systemic energy balance and metabolic homeostasis. The inherent metabolic flexibility of skeletal muscle, which is its ability to shift between energy substrates according to physiological status and substrate availability, allows for optimal energy supply and adaptation to different exercise intensities (1). An excessive buildup of intramyocellular lipids (IMCLs) can, however, impair this metabolic flexibility in skeletal muscle. This accumulation also interferes with glycogen utilization and reduces substrate-switching efficiency. Therefore, such disruptions negatively affect exercise performance. They are also regarded as contributors to the development of metabolic syndromes such as obesity, insulin resistance, and type 2 diabetes. An intriguing phenomenon regarded as the “athlete’s paradox” demonstrates that individuals engaged in long-term, regular training demonstrate efficient fatty acid (FA) esterification into triglycerides (TGs). These TGs are then stored in lipid droplets (LDs), which appear inert, even when IMCL levels might be high. Regulated LD dynamics and lipolysis accompany this process. These mechanisms promote lipid turnover and lessen the inhibitory effect of lipids on insulin sensitivity (2, 3). The existence of this phenomenon points to exercise training as a potent intervention for improving lipid metabolic dysregulation. It can also enhance metabolic flexibility, especially for individuals with obesity (4, 5).

IMCLs are primarily stored in the form of LDs. LDs are not merely simple, inert organelles; instead, they are dynamic structures. Their composition includes a neutral lipid core, a surrounding phospholipid monolayer, and a variety of LD-associated proteins (LRPs) (6). Over 300 LRPs have been identified through proteomic studies (7). These proteins play critical roles in regulating the dynamic processes of LDs, including their formation, fusion, storage, mobilization, and degradation, and they also respond to the cell’s energy status (8–13). The Perilipin (PLIN) family stands out among these LRPs as the most extensively researched group. Five members, designated PLIN1-5, constitute this family, and they each exhibit different tissue expression patterns. Adipose tissue demonstrates predominant enrichment of PLIN1 and PLIN4; whereas, high expression levels of PLIN2, PLIN3, and PLIN5 are identified in skeletal muscle across various species (14). For explaining the regulatory mechanisms governing lipid metabolism in skeletal muscle, a thorough understanding of the functions of PLIN2, PLIN3, and PLIN5 is essential.

Complex communication and cooperation among subcellular organelles form the basis for the accurate regulation of intracellular lipid metabolism. In recent years, significant research interest has focused on membrane contact sites (MCSs) (15, 16). These sites function as platforms facilitating direct material exchange (e.g., lipid transport) and signal transduction between different organelles. The definition of MCSs involves regions where organelle membranes are in close apposition (typically 10-30 nm). Specific protein complexes mediate this proximity. Facilitating the rapid, non-vesicular transport of molecules such as lipids and ions is a key function of these sites. They also offer critical contributions to organelle membrane integrity and lipid homeostasis and hold the ability to be dynamically remodeled according to cellular metabolic demands (16). Instead of existing in isolation, LDs form extensive MCSs with a variety of organelles. These include the endoplasmic reticulum, mitochondria, and lysosomes. Central regulatory functions in LD biogenesis, growth, and lipolysis are performed by these contact sites. In addition, they affect the ensuing fate of FAs, including their transport into mitochondria for β-oxidation (17–19). A crucial finding is that exercise training can remodel both the structure and function of inter-organellar MCSs. This remodeling enhances cellular homeostasis and leads to improved insulin sensitivity (20).

PLIN proteins, functioning as key LRPs, anchor to the LD surface and directly influence LD dynamics and function. Since LDs also engage in extensive interactions with other organelles through MCSs, it is hypothesized that PLIN proteins play a critical role in regulating exercise-induced improvements in skeletal muscle lipid metabolism through the mediation of these LD-organelle interactions. A study of PLIN protein mediation of interactions between LDs and other organelles can be carried out in this review. Besides, the review explores the molecular mechanisms that explain their contributions to exercise-regulated lipid homeostasis in skeletal muscle. The objective of this review is to shed light on the molecular mechanisms responsible for the health benefits of exercise in enhancing metabolic disorders. It also intends to offer a theoretical framework for the development of targeted therapeutic strategies for metabolic conditions such as obesity and diabetes. We performed a systematic literature search of the PubMed, Web of Science, and Scopus databases for relevant articles published up to August 2025. The search strategy combined keywords and MeSH terms, including “perilipin,” “PLIN,” “lipid droplet,” “organelle interaction,” “skeletal muscle,” “exercise,” “endurance training,” “high-intensity interval training,” “lipid metabolism,” and “lipophagy.” We included original research (both in vivo and in vitro studies), reviews, while excluding non-English articles and conference abstracts without full-text. The selection process focused on literature elucidating the roles of PLIN proteins (specifically PLIN2, PLIN3, and PLIN5) in skeletal muscle, their interactions with organelles, and their regulation by different exercise modalities.

## 2 Role of PLIN proteins in skeletal muscle lipid metabolism

Found on the surface of LDs, PLINs function as critical regulatory proteins. Their role is critical in the regulation of lipid storage in adipocytes. A tight regulation of the balance between TG synthesis and breakdown is exerted by these proteins. This regulation occurs in response to phosphorylation changes that are induced by hormonal signals such as epinephrine and insulin (21, 22). Direct modulation of the LD degradation rate is another function of PLINs. They achieve this by controlling lipase access and activity (e.g., adipose triglyceride lipase (ATGL) and hormone-sensitive lipase (HSL)) at the LD surface. Sequence similarity is a shared characteristic among the five members of the PLIN family. The conserved N-terminal PAT domain (PLIN, PLIN2, PLIN3 domain) is essential for their binding to LDs. The C-terminal regions of these proteins also hold hydrophobic sequences responsible for stabilizing their localization on the LD surface. Besides, they are capable of facilitating LD dynamics. In specific regions of PLIN members, different subcellular localizations, characteristics, and functional roles in processes (e.g., LD formation, lipolysis, fatty acid oxidation, and overall metabolic regulation may occur. For instance, C-terminal regions of PLIN1 and PLIN4 are defined by an extended structure and an enrichment of hydrophobic amino acids that promote their stable attachment to the LD surface. Moreover, in the C-terminus of PLIN4, it is also identified that highly repetitive sequences exist. These sequences likely mediate its preferential binding to cholesterol esters (19). PLIN2, PLIN3, and PLIN5 exhibit diverse structural and functional characteristics. Accordingly, such particular PLINs exhibit high expression in skeletal muscle. The C-terminal region of PLIN2 is represented by multiple repeat sequences. Its stability relies on its association with LDs; thus, upon LD degradation, PLIN2 is subjected to ubiquitin-proteasome-mediated degradation in the cytoplasm (22, 23). The composition of PLIN3 includes several hydrophilic amino acids. This protein contributes to forming and stabilizing nascent LDs and, specifically, maintains its stability in the cytoplasm (21, 24, 25). PLIN5 exhibits a unique structure. Specifically, its C-terminus is enriched with hydrophobic domains and includes a mitochondrial targeting sequence. This configuration enables PLIN5 to interact with both LDs and mitochondria, leading to bidirectional regulation of LD synthesis and degradation (26). Owing to this structural and functional diversity, PLIN family members can act in synergy. They are also able to respond to a variety of metabolic signals and dynamically regulate lipid metabolism. For a clear understanding of lipid metabolism regulation and its implications in metabolic disorders, a detailed analysis of the structure-function relationships in the PLIN family is crucial. A focus on the specific functions of PLIN2, PLIN3, and PLIN5 will be offered in the sections that follow. These three perilipins are the ones primarily expressed in skeletal muscle.

### 2.1 Role of PLIN2 in LD formation and stability

PLIN2 functions as a key protein in skeletal muscle, where it governs the formation of LDs and maintains their structural stability. Stable binding to the surface of nascent LDs is facilitated by its N-terminal PAT domain through hydrophobic interactions. This binding mechanism ensures the stability of both the PLIN2 protein itself and the integrity of the LDs, while supporting LD biogenesis, growth, and maturation. A close correlation between PLIN2 expression levels and the abundance of LDs in skeletal muscle has been confirmed by multiple studies. PLIN2 content demonstrates a positive relationship with IMCL content in the skeletal muscle of both rodents and humans (27), moreover, its expression levels are higher in females and in type I muscle fibers (19, 28). Functionally, LDs are regulated by PLIN2 acting as a barrier. PLIN2 restricts the access of lipolytic enzymes such as ATGL and HSL to the TG core of LDs through interactions, which then inhibits basal lipolysis (26, 29). The accumulation of IMCL increases when PLIN2 is overexpressed in skeletal muscle cells, leading to LDs that are larger and more numerous (19). Following weight loss or metformin treatment in individuals with insulin resistance, an increase in muscle PLIN2 content enhances insulin sensitivity; this likely acts as a compensatory mechanism promoting lipid storage as TGs to alleviate lipotoxicity (26). Upregulation of PLIN2 expression, however, is not beneficial under all circumstances. Pathological states involving lipid overload, illustrated by models of amyotrophic lateral sclerosis, exhibit increased PLIN2 expression along with the downregulation of the lipolytic enzyme ATGL and the mitochondrial fatty acid oxidation enzyme carnitine palmitoyltransferase 1A (CPT1A), resulting in disrupted lipid metabolism, mitochondrial dysfunction, and intramyocellular lipotoxicity (30). A crucial distinction can be observed between PLIN2 in skeletal muscle and PLIN1 in adipocytes: skeletal muscle PLIN2 in skeletal muscle is minimally phosphorylated in response to lipolytic stimuli, such as epinephrine or muscle contraction (31). In addition, *in vitro* analyses demonstrated that treating C2C12 myotubes with oleic acid upregulates PLIN2 expression dose- and time-dependently and also enhances LD accumulation; whereas, knocking down PLIN2 results in an increase in LD volume and TG hydrolysis activity (32). Therefore, skeletal muscle cells exhibit a reciprocal stabilizing relationship between PLIN2 and LDs. PLIN2 primarily functions to enhance IMCL storage and LD stability through the inhibition of basal lipolysis, reducing the toxicity associated with excess fatty acids, while it might worsen metabolic dysfunction during conditions of extreme lipid overload.

### 2.2 Role of PLIN3 in LD biogenesis and lipid metabolism regulation

PLIN3 demonstrates wide expression across various tissues. It was identified as the first PLIN family member capable of dynamic translocation between the cytoplasm and LDs, a process critically contributing to the initial biogenesis of LDs. In its N-terminal PAT domain, a conserved aspartate residue (E86) interacts with diacylglycerol (DAG). This interaction targets PLIN3 towards DAG-rich areas of the endoplasmic reticulum membrane and nascent LDs, thereby facilitating their formation and stabilization. Therefore, PLIN3 acts as a recognized marker for early LD formation. Other PLIN proteins, including PLIN2, gradually replace PLIN3 as LDs mature; cells utilize this dynamic replacement mechanism to adapt LD properties and functions according to metabolic demands (33). Recent studies have also indicated a critical function for PLIN3 in lipophagy. In this process, it acts as a substrate for chaperone-mediated autophagy (CMA). CMA-targeting motifs present in PLIN3 are recognized by heat shock protein 70 (Hsc70), which facilitates its translocation into lysosomes for degradation. The stability of the LD surface is compromised by the removal of PLIN3, rendering LDs more susceptible to degradation by either ATGL or proteins related to macroautophagy (34). Intracellular lipid homeostasis is maintained, and excessive lipid accumulation is prevented through this mechanism.

While PLIN3 expression is confirmed in human skeletal muscle (14, 35), specific functional studies focusing on this tissue are still limited. Increased PLIN3 expression levels are observed in females’ skeletal muscle; however, exercise training does not significantly change these levels (14). A specific study involving obese individuals indicated a significant finding: notwithstanding similar expression levels of key enzymes in TG synthesis or lipolysis, the protein abundance of PLIN3 (relative to IMCL content) was lower in the skeletal muscle of obese participants compared to lean controls (36). This suggests a potential connection between PLIN3 and lipid metabolic dysfunction related to obesity. Regarding its cellular location, PLIN3 is present on both the LD surface and in the cytoplasm. Its distribution in isolated soleus muscle fibers remains largely unchanged by either muscle contraction or epinephrine stimulation (37). PLIN3 detection has also occurred in skeletal muscle mitochondria, though acute contraction does not lead to significant changes in its abundance (26); the specific function it performs in mitochondria is currently unclear.

### 2.3 Bidirectional regulatory role of PLIN5 in LD formation and lipolysis

In skeletal muscle lipid metabolism, PLIN5 holds a critical and multifaceted role, especially concerning the regulation of lipid storage and oxidation. PLIN5 expression is high in tissues with high oxidative capacity, such as skeletal muscle, heart, and liver (38), the protein holds unique structural features; specifically, its C-terminus harbors a mitochondrial targeting sequence. This sequence enables PLIN5 to act as a structural link connecting LDs and mitochondria. Therefore, PLIN5 can exert a unique bidirectional regulatory effect on LD formation, storage, and lipolysis (31).

Concerning its function in promoting lipid storage, upstream signaling pathways accurately regulate PLIN5 expression. These pathways include the activation of peroxisome proliferator-activated receptors (PPARs), particularly PPARα, by FFAs (38). FFAs act as ligands for PPARs, activating these transcription factors. Activated PPARs, accordingly, induce the expression of the PLIN5 gene, leading to increased PLIN5 protein levels. High PLIN5 then promotes the esterification of excess FFAs into TGs and their following storage in LDs. This suggests that PLIN5 might indirectly influence LD formation through its effect on cellular FFA uptake. For instance, experiments modulating PLIN5 expression levels (via overexpression or knockdown) in cardiomyocytes demonstrated regulation of the expression and activity of fatty acid translocase cluster of differentiation 36 (CD36) and lipoprotein lipase (LPL), respectively (39, 40). Besides, PLIN5 coats the LD surface, forming a physical barrier. This barrier restricts lipase access to substrates under basal conditions, which promotes TG accumulation and strengthens LD stability. A proposed mechanism suggests PLIN5 could partially inhibit basal FA β-oxidation by modulating mitochondrial spatial distribution or interacting with FA metabolic enzymes, thus prioritizing energy storage in LDs (41). These combined mechanisms highlight its protective function in facilitating lipid storage.

Conversely, the role of PLIN5 in controlling lipolysis and fatty acid utilization displays inherent bidirectionality. Under basal or unstimulated states, PLIN5 binds to comparative gene identification-58 (CGI-58), which is an activator of ATGL. This binding prevents CGI-58 from interacting with ATGL (42). The outcome is suppression of the basal lipolytic rate, protecting cells against potential lipotoxicity arising from excessive FFA release. However, cellular energy demands increase, or hormonal signals such as β-adrenergic agonists stimulate the cells, activating protein kinase A (PKA). PKA subsequently phosphorylates PLIN5. Phosphorylation induces a conformational change in PLIN5, leading to the release of CGI-58. Released CGI-58 then binds to and activates ATGL, initiating efficient lipolysis. Stored TGs are broken down into FFAs and glycerol for mitochondrial oxidation through this process (42, 43). The PLIN5-mediated physical connection between LDs and mitochondria is crucial here; it likely facilitates the efficient, targeted movement of FFAs released from LDs directly to adjacent mitochondria for β-oxidation to facilitate integration of lipid storage and energy supply.

Recent studies have indicated a critical role for PLIN5 in lipophagy. Identification of PLIN5 as a selective substrate for CMA has been established (44–46). Its protein sequence is predicted to contain KFERQ-like motifs recognizable by the cytosolic chaperone Hsc70. Hsc70 binds PLIN5 and delivers it to the lysosomal-associated membrane protein 2A (LAMP2A) receptor situated on the lysosomal membrane, facilitating its entry into the lysosomal lumen for degradation (47). Regulation of LD turnover and the maintenance of cellular lipid homeostasis rely significantly on the CMA-mediated degradation of PLIN5. Impaired PLIN5 degradation due to CMA dysfunction can cause excessive LD accumulation and disrupt lipid metabolic homeostasis. Such dysregulation carries significant pathological implications for metabolic disorders, non-alcoholic fatty liver disease being one example (47). However, it is necessary to further explain the following two aspects: the full extent of PLIN5-mediated lipophagy’s role in skeletal muscle and its physiological importance for exercise adaptation and metabolic diseases.

In conclusion, PLIN5 in skeletal muscle functions beyond being merely an LD coating protein. In effect, it represents a regulatory hub where lipid storage and mobilization are dynamically controlled according to metabolic signals. Moreover, its unique structure enables functional coupling between LDs and mitochondria that enables fatty acids to be efficiently utilized as necessary. Therefore, in skeletal muscle, it is essential to maintaining energy homeostasis and metabolic flexibility.

## 3 Regulation of PLINs-mediated organelle interactions and lipid metabolism in skeletal muscle by exercise

Intracellular energy homeostasis is crucially sustained through inter-organellar interactions. The dynamic equilibrium between LD synthesis and degradation also depends on these interactions, particularly the physical and functional connections established through MCSs. For an extended period, researchers regarded LDs merely as inert intracellular depots for lipid storage. However, a growing body of evidence presently indicates that LDs function as highly dynamic organelles. They actively participate in a range of crucial cellular processes, which include lipid metabolism, energy homeostasis, membrane biosynthesis, and cellular signal transduction (48). Lipid uptake in skeletal muscle, for instance, relies primarily on a collection of membrane transport proteins. This group includes fatty acid-binding proteins (FABPs), fatty acid transport proteins (FATPs), and CD36 (49–51). Insulin stimulation prompts FATP1/4 translocation, while muscle contraction induces CD36 translocation, both moving these proteins to the plasma membrane or transverse-tubule membrane. The result of this translocation is an enhancement in FA uptake efficiency. In addition, CD36 expression levels increase following a high-fat diet, a change that further boosts FA uptake (51). Once FAs enter myocytes, their metabolic fate is determined by the cell’s energy demands and overall metabolic status. FAs are preferentially esterified into TGs in the ER under resting conditions. Then, these TGs are stored as LDs, representing an alternative fate to direct channeling towards mitochondrial oxidation; whereas, cells mobilize FAs from LDs through efficient degradation pathways when encountering increased energy demands, such as during exercise or starvation. This mobilization process involves the formation of MCSs connecting LDs with other essential organelles such as mitochondria, the ER, and lysosomes. Lipid metabolism is effectively “channeled” through two primary mechanisms, lipolysis and lipophagy. This ensures the accurate transport of FAs to the locations where energy production occurs (2, 16, 17). Specific intermembrane multi-protein complexes, for instance, lipid transfer proteins (LTPs) or ion channels, typically make up LD-associated MCSs. Rapid and selective exchange of lipids or ions between organelles is mediated by these complexes; this represents a fundamental mechanism for maintaining the lipid composition of organellar membranes and overall cellular lipid homeostasis (2). Members of the PLIN protein family play critical roles in these dynamic interactions. These proteins perform dual functions: they coat the LD surface and are also directly involved in mediating LD contact with various organelles. Through these actions, they achieve spatiotemporal regulation of lipid storage and mobilization responding to cellular metabolic needs, stress situations, or environmental shifts (2). Current research suggests PLIN3 is mainly involved in LD biogenesis occurring at the ER. However, a definitive establishment of its direct role in facilitating LD contact with other organelles is still pending. In contrast, PLIN2 and PLIN5 exhibit more extensive involvement in modulating the dynamic interactions between LDs and other cellular compartments, including mitochondria and lysosomes.

The expression, localization, and activity of PLIN proteins in skeletal muscle are significantly modulated by exercise training. Regulation of intramuscular triglyceride (IMTG) storage, lipolysis, and interactions with organelles such as mitochondria occurs through these changes, thereby mediating the adaptive changes in skeletal muscle lipid metabolism induced by exercise. This chapter will systematically elucidate how exercise influences PLINs, thereby reshaping the dynamic interactions between LDs and the endoplasmic reticulum, mitochondria, and lysosomes, ultimately promoting efficient lipid utilization and metabolic health. The regulation of PLINs mediated by exercise, organelle interactions, and lipid metabolism are summarized in **Table 1**.

**Table 1:** Exercise-mediated regulation of PLINs, organelle interactions, and lipid metabolism.

PLINs	Exercise type	Expression level	Localization	Mitochondrial function	Lipophagy	FAO	LDs changes	Reference
PLIN2	AE	No significant change	LDs, Preferential consumption of PLIN2-coated LDs	-	-	↑	↓ Large LDs ↑ Small LD density	(57, 70)
	MICT	↑	-	↑ Biogenesis, stabilized LD-mitochondria contacts	↑ (Liver)	↑	IMTG breakdown, ↓ Ectopic lipid deposition	(58, 67, 68, 112)
	HIIT	-	-	-	-	↑	-	-
PLIN3	AE	↑	-	-	-	↑	-	(71)
	MICT	↑	Mitochondrial localization in oxidative fibers	↑ FAO capacity ↑Mitochondrial Biogenesis	-	↑	↓ Ectopic lipid deposition	(72)
	HIIT	-	-	-	-	-	-	-
PLIN5	AE	No significant change	LD-mitochondria contact sites	↑ FAO	-	↑	IMTG breakdown	(90)
	MICT	No significant change	LDs Mitochondria	↑ Biogenesis, stabilized LD-mitochondria contacts	-	↑	IMTG breakdown ↑ FAO	(58, 90)
	HIIT	↑	↑ LD-mitochondria contact sites	↑ Mitochondrial mass and function	-	↑	↓ Ectopic lipid deposition ↑ FAO	(61, 92)

The regulation of PLIN2-mediated lipophagy by MICT has been studied in the liver, whereas other studies, unless specified otherwise, were conducted in skeletal muscle. AE, Acute Exercise; MICT, Moderate-intensity continuous training; HIIT, High-intensity interval training; FAO, Fatty acid oxidation; LD, Lipid droplets; IMTG, Intramuscular triglyceride; MAMs, Mitochondria-associated membranes; ↑, Increase; ↓, Decrease; -, None.

### 3.1 Overall adaptation of skeletal muscle lipid metabolism to exercise and the general response of PLINs

Reducing systemic and skeletal muscle lipid dysregulation can be achieved through regular physical exercise, an established non-pharmacological strategy. Skeletal muscle acts as a primary metabolic tissue and has remarkable plasticity. Therefore, it is subjected to extensive structural and functional adaptations when responding to contractile activity. The muscle’s efficiency in utilizing energy substrates, particularly FAs, is enhanced by these adaptations; this helps meet ATP demands and enhance fatigue resistance. Significant reductions in IMTG levels and activation of lipolytic and lipid oxidation pathways can occur after a single bout of acute exercise, as studies demonstrate, offering immediate metabolic benefits for individuals with obesity and type 2 diabetes mellitus (T2DM) (52). Nevertheless, achieving sustained health benefits requires chronic adaptations induced by long-term, regular exercise, which include increased expression of LRPs and lipases (53, 54). Moderate-intensity continuous training (MICT) is traditionally recognized as an effective method for enhancing FA oxidation capacity and metabolic flexibility in obese individuals. Overall metabolic efficiency can be enhanced through MICT as it promotes mitochondrial biogenesis and oxidative capacity in skeletal muscle. MICT upregulates key lipolytic protein expression in skeletal muscle while also activating mitochondrial fatty acid oxidation (FAO)-related enzyme activity. Facilitating IMTG breakdown and FA utilization are key actions, which attenuate the accumulation of harmful lipid intermediates such as DAG and ceramides; finally, insulin sensitivity and skeletal muscle lipid homeostasis are enhanced (53).

HIIT has received significant recognition in recent years concerning its role in improving metabolic health, attributed to its time efficiency and distinct physiological effects. Similar or superior outcomes compared with traditional MICT can be achieved with HIIT in metabolic disease interventions, according to multiple studies (55). This establishes HIIT as a promising approach for health promotion and disease rehabilitation. Improvements in blood lipid profiles, enhanced insulin sensitivity, reduced ectopic lipid deposition in hepatic and muscle tissues, and promotion of insulin signal transduction result from HIIT (56). Besides, the phenomenon of HIIT-induced excess post-exercise oxygen consumption (EPOC), often called the afterburn effect, might contribute to long-term body fat regulation. The underlying mechanisms for HIIT-mediated lipid metabolism improvements consist of multiple pathways. These include promoting mitochondrial biogenesis, enhancing mitochondrial function, and ameliorating inflammatory status. Notwithstanding the well-documented effectiveness of HIIT in enhancing lipid metabolism and its increasing use as a primary exercise intervention, significant unresolved scientific questions remain. Specifically, the molecular mechanisms governing its regulation of skeletal muscle LD utilization—especially concerning organelle interactions and the accurate roles of LD-binding proteins (PLINs)—require further research.

Explaining how PLIN family members, as key regulatory proteins on the LD surface, respond to different exercise modalities and contribute to exercise’s metabolic benefits is a major focus of current research.

As key regulatory proteins on the surface of LDs, members of the PLINs family respond to exercise stimuli through their expression, localization, and functional states, serving as critical mediators in the adaptation of skeletal muscle lipid metabolism induced by exercise. Skeletal muscle PLIN protein expression is more strongly regulated by long-term, regular exercise (e.g., MICT and HIIT). In contrast, single, acute exercise bouts produce minimal or inconsistent effects. Bajpeyi et al. reported a decrease in total skeletal muscle lipid content after acute endurance exercise; this was represented by more small LDs and fewer large LDs in the sarcoplasm; however, no significant changes were observed in PLIN2 or PLIN5 protein levels (57). It is noteworthy that during acute exercise, LDs coated with PLIN2 and PLIN5 are preferentially consumed, indicating their critical role in regulating immediate lipid mobilization. Interestingly, after six weeks of training, the preferential utilization of PLIN5-coated LDs persists, while the preference for PLIN2-coated LDs diminishes, suggesting distinct adaptive roles. Furthermore, the content of PLIN2 and PLIN5 in skeletal muscle fibers of athletes undergoing long-term endurance training is significantly higher than in sedentary controls (58). Reinforcing these findings, a recent study confirmed that PLIN5 protein levels are elevated in endurance-trained human subjects and directly correlate with both muscle oxidative capacity and whole-body insulin sensitivity. This highlights PLIN5’s role not just in lipid storage but as a marker of metabolic health. Mechanistically, this study further revealed that PLIN5 plays a dual role: under basal conditions, it limits lipolysis, thereby protecting against lipotoxicity; however, during contraction-mediated stimulation, it paradoxically enhances fatty acid oxidation, ensuring an efficient supply of fatty acids to mitochondria (59). This aligns with observations of a positive correlation between PLIN5-associated LDs and maximal oxygen consumption (VO_2max_) in athletes (14, 60), suggesting PLIN5 is crucial for finely tuning lipid supply to meet oxidative demand during exercise. Different training modalities can also fine-tune this adaptation. Sirago et al., however, compared long-term MICT versus HIIT in individuals with obesity and found that only HIIT significantly enhanced skeletal muscle mitochondrial mass, function, and PLIN5 expression (61). These findings collectively suggest that members of the PLIN family may play complex and critical roles in exercise-induced skeletal muscle IMTG mobilization, enhanced fatty acid oxidation, and long-term metabolic adaptations.

### 3.2 PLINs-mediated LD-ER interactions and LD biogenesis

LD biogenesis begins at the ER membrane where cytosolic glycerol-3-phosphate is first converted to DAG. Then, through a reaction catalyzed by either diacylglycerol acyltransferase (DGAT) 1 or 2, DAG is esterified to TGs. Inside the hydrophobic core of the ER bilayer, newly synthesized neutral lipids (incl. TGs and cholesterol esters) accumulate and form a “lipid lens” structure. This structure eventually buds towards the cytosol, developing into mature LDs. Diverse LD-associated proteins are recruited to the surface of these nascent LDs throughout this entire process. These proteins are classified into two groups. The first group contains proteins such as DGAT1, which translocate from the ER membrane onto the surface of mature LDs. They have hairpin-like membrane anchor structures enabling bidirectional lipid transport. The second group features proteins with amphipathic helix (AH) domains. These proteins associate tightly with the LD monolayer surface. They are diffusely distributed in the cytosol when LD formation is absent but are selectively recruited upon LD biogenesis. The PLIN family of proteins exemplifies this second category.

Studies confirm that PLIN3 and PLIN5 localize to both LDs and the ER (62, 63). Intracellular neutral lipid accumulation is promoted by the heterologous expression of PLINs, which also induces LD formation (64). Anchoring PLINs to the ER membrane, achieved through fusion with ER membrane proteins, notably facilitates the redistribution of the ER membrane around LDs. This results in close proximity between the ER and LDs (65). Unexpectedly, expressing a PLIN3 chimeric protein anchored to the ER membrane in cells lacking LDs caused ER membrane expansion and vesiculation. This effect was particularly noticeable in the perinuclear membrane, leading to crescent-shaped membrane domains with LD-like characteristics. Enrichment of DAG and key ER proteins crucial for early LD biogenesis, such as Seipin and Peroxin30 (Pex30), was observed in these domains. These domains transformed into nascent LDs upon the induction of neutral lipid synthesis. Moreover, the formation of these specialized domains was dependent on intracellular DAG levels. *In vitro* experiments further confirmed PLIN3’s binding to DAG-containing liposomes, suggesting PLIN3 might initiate LD formation through sensing or enriching DAG (66).

The expression level of PLIN2 in skeletal muscle demonstrates a close association with lipid storage capacity. For instance, higher PLIN2 content is identified in oxidative muscle fibers compared to glycolytic muscle fibers, and significant co-localization exists between PLIN2 and LDs (67). Deng et al. reported findings related to lipid accumulation in C2C12 myotubes. Their work demonstrated that PLIN2, a major LD surface protein, enhances TG accumulation and LD growth. It achieves this by recruiting and anchoring the regulatory protein Rab18 to the LD surface, thereby forming a complex with Acyl-CoA long-chain synthetase 3 (ACSL3). However, the knockout of PLIN2 in C2C12 cells led to several changes: increased contacts between LDs and mitochondria, a reduced number of LDs despite an increased overall volume, and impaired mitochondrial activity (68). Taken together, these findings indicate that PLINs not only preserve LD stability but also perform a critical function during the initial stages of LD biogenesis.

Although PLINs play a critical role in endoplasmic reticulum-mediated LD biogenesis, research on whether and how exercise affects the expression, recruitment, or function of PLINs at ER-LD contact sites, thereby influencing LD biogenesis and size, remains relatively scarce. Future studies could investigate whether exercise modulates the transcription, translation, or modification of PLIN3/2, impacting the formation rate of nascent LDs and the overall dynamics of the LD pool. Additionally, whether exercise-induced ER stress or the unfolded protein response (UPR) affects PLINs function or ER-LD interactions represents a research gap worthy of further exploration.

### 3.3 Exercise-regulated PLINs-Mediated LD-Mitochondria interaction and fatty acid oxidation

The efficient transport of FAs from LDs to mitochondria for fueling β-oxidation represents a critical facet of lipid metabolism. Early studies utilizing methods such as electron microscopy identified close, transient physical interactions occurring between LDs and mitochondria. These interactions were observed in tissues with high oxidative activity, such as skeletal muscle, liver, and adipose tissue. Gemmink et al. employed super-resolution microscopy for an in-depth analysis of PLIN distribution on the LD surface in skeletal muscle. Their results indicated that PLIN2 and PLIN5 do not create a continuous lipolytic barrier, contrary to previous postulations. Instead, these proteins are distributed in punctate, heterogeneous patterns that might offer discrete binding locations for molecules such as lipases. Crucially, PLIN2 and PLIN5 generally do not co-localize in the same LD region; they are localized to adjacent positions instead. This observation supports their distinct functions in regulating lipolysis under both basal and stimulated conditions (69).

Regarding the impact of exercise on PLIN2/3 in LD-mitochondria interactions, Shepherd found that PLIN2-coated LDs are selectively consumed during acute exercise, but this preference diminishes after long-term training (70). An increase in PLIN3 protein levels in human skeletal muscle following one prolonged endurance exercise session was reported by Covington et al.; this increase correlated with *in vitro* palmitate oxidation rates and whole-body fat oxidation rates (71). Ramos et al. found that long-term endurance training high mitochondrial PLIN3 levels in the oxidative muscle fibers of a rat model. However, acute muscle contraction through electrical stimulation did not change mitochondrial PLIN3 levels (72). These results indicate PLIN3 contributes to the exercise-induced enhancement of fat oxidation capacity, mainly through chronic adaptation or changes in specific subcellular compartments. Furthermore, some studies have also confirmed that the role of PLIN2 in regulating the dynamic interactions between LDs and mitochondria is complex and dynamic. Research conducted by Bosch et al. demonstrated that PLIN2 expression on macrophage LDs increases during immune stress responses triggered by bacterial lipopolysaccharides (LPS), while PLIN5 expression decreases. This reduction in PLIN5 facilitated the detachment of LDs from mitochondria. Therefore, a metabolic shift occurred, changing the primary cellular substrate from glucose to FAs. This suggests that a similar dynamic interplay between PLIN proteins could potentially regulate metabolic profiles in skeletal muscle under different physiological conditions (73). Other studies offer further insights. Under conditions of glucose deprivation, phosphofructokinase liver (PFKL) type, a key glycolytic enzyme, is phosphorylated, resulting in decreased activity. Then, phosphorylated PFKL binds to and phosphorylates PLIN2. Phosphorylated PLIN2 then binds to CPT1A located on the outer mitochondrial membrane. This binding facilitates the anchoring of LDs to mitochondria and the recruitment of ATGL to the contact interface, thereby initiating fat mobilization in hepatocytes (74). A similar phosphorylation-dependent mechanism may also exist in skeletal muscle. In addition, analyses into aflatoxin B1 (AFB1)-induced hepatic lipotoxicity indicated an enhanced interaction between mitochondria-localized p53 and LD-localized PLIN2 due to AFB1. This enhancement increased LD-mitochondria contact. However, LDs under these specific conditions are primarily degraded through the lysosome-dependent lipophagy pathway. This leads to abnormal accumulation of intracellular lipids; whereas, targeted inhibition of either p53 or PLIN2 was observed to activate lipophagy, promote the degradation of LDs, and alleviate lipotoxicity (75). In pancreatic β-cells, the absence of PLIN2 causes excessive FA release through the lysosomal pathway. These FAs then accumulate anomalously in mitochondria, precipitating mitochondrial damage and impairing glucose-stimulated insulin secretion (GSIS). Moreover, similar aberrant FA flux towards mitochondria and the ensuing mitochondrial damage have been observed under conditions of nutrient overload, including increased levels of glucose and lipids. These observations imply that PLIN2 acts as a critical regulator directing FA trafficking from LDs towards unique organelles, such as mitochondria or lysosomes (76). Recent research identified the liver-derived exercise factor fibroblast growth factor 21 (FGF21) as a lipid metabolism regulator. Studies demonstrate that FGF21 levels are modulated by exercise intensity, with high-intensity exercise eliciting significantly elevated serum FGF21 concentrations compared to moderate-intensity exercise (77). While chronic high-intensity training reduces FGF21 mRNA expression, it enhances FGF21 signaling sensitivity through upregulation of FGF21 receptor expression, thereby mitigating lipid metabolism dysregulation (78). The interplay between PLIN5 and FGF21 is particularly noteworthy. For instance, Jia et al. reported correlated high expression levels of FGF21 and PLIN5 in the liver and skeletal muscle of obesity-resistant mice, whereas PLIN2 expression was reduced (79). Providing direct evidence for this link, a study using a skeletal muscle-specific PLIN5 overexpressing mouse model (MCK-Plin5) demonstrated that upregulating PLIN5 alone was sufficient to drive an 80-fold increase in muscle FGF21 gene expression and elevate serum FGF21 levels. This muscle-derived FGF21 conferred systemic metabolic protection, including reduced hepatic inflammation and enhanced adipose tissue “browning,” highlighting a myokine-mediated mechanism originating from fast-twitch fibers (80). Although the precise mechanisms underlying exercise-induced FGF21 activation remain unclear, prior studies have established that high-intensity training markedly increases hepatic free fatty acid (FFA) levels and depletes glycogen stores, driving FGF21 secretion in the liver through PPARα and ATF4 signaling pathways (81, 82). In skeletal muscle, exercise stimulates FGF21 expression via activation of the AMP-activated protein kinase (AMPK) and Akt signaling cascades (83). Collectively, these findings suggest that exercise regulates lipolysis and lipid oxidation while suppressing lipid accumulation through the AMPK/Akt-FGF21-PLINs axis (79). This work offers novel perspectives on the role of exercise in modulating skeletal muscle lipid metabolism. In summary, the role of PLIN2 in LD-mitochondria interactions is complex; it includes promoting contact to facilitate mitochondrial oxidation and regulating the lipophagy pathway, with its specific function being dependent on the cell type and metabolic state.

Current research presents inconsistent, and occasionally contradictory, findings regarding PLIN5’s specific role in oxidative tissues. Reduced LD content alongside increased FA oxidation capacity is demonstrated in the cardiac and skeletal muscle of PLIN5 gene knockout (PLIN5-KO) mice (40, 41, 84, 85); whereas, LDs accumulation and enhanced LD-mitochondria interactions result from heart-specific PLIN5 overexpression (86). PLIN5-KO mice exhibit contrasting effects in the liver, indicating reduced LD-mitochondria interactions coupled with decreased FA oxidation capacity (84). Opposite results are seen with PLIN5 overexpression; in the skeletal muscle of rats fed a high-fat diet or mouse brown adipose tissue under cold stress, this enhances oxidative gene expression and promotes mitochondrial cristae formation (87, 88). These observations collectively suggest that multiple factors modulate PLIN5’s function in oxidative tissues, including the specific cell type, the prevailing metabolic state, and other regulatory mechanisms. One hypothesis hypothesizes that PKA phosphorylation of PLIN5 acts as a key regulator. This phosphorylation event may determine whether PLIN5 facilitates LD biogenesis or promotes catabolism. Zhang et al. put forth a model where, under basal conditions, PLIN5 interacts with CGI-58 (a co-activator of ATGL), thereby inhibiting it. PKA-mediated phosphorylation of PLIN5, triggered by stress conditions such as cold exposure, fasting, or exercise, causes the release of CGI-58, which then promotes ATGL-mediated lipolysis (89). However, MacPherson et al. observed basal phosphorylation of PLIN3 and PLIN5 under resting conditions; however, PLIN2 phosphorylation was undetectable. Besides, serine phosphorylation levels for PLIN3 and PLIN5 did not change significantly when responding to epinephrine stimulation or muscle contraction-induced lipolysis. Interactions occur between PLIN2, PLIN3, PLIN5 and the lipases HSL and ATGL; however, these interactions displayed no significant changes following lipolytic stimulation (31). Based on these findings, the regulation of lipolysis in skeletal muscle induced by exercise or adrenergic stimulation appears not primarily dependent on phosphorylation state changes in PLIN3 and PLIN5. Other regulatory mechanisms are thus likely involved, such as changes in protein interaction networks or subcellular localization.

PLIN5 is a LD-associated protein expressed mainly in oxidative tissues such as skeletal and cardiac muscles; it plays a central role in regulating skeletal muscle lipid metabolism during exercise. Super-resolution microscopy analyses indicate PLIN5 localization extends beyond the surface of cytosolic LDs; it is also identified in regions proximal to mitochondria and associated with LD-mitochondria contact points. This localization supports a role for PLIN5 in facilitating FA release from LDs and their channeling toward mitochondrial oxidation (69). PLIN5 serves as a bridging protein between LDs and mitochondria, interacting with mitochondrial outer membrane proteins through its C-terminal domain to regulate lipid storage and mobilization.

Exercise significantly modulates PLIN5’s expression, its subcellular localization, and the organelle interactions it facilitates. Rapid mobilization of PLIN5 occurs during acute exercise to promote lipid metabolism. A significant decrease in IMTG levels after 30 minutes of electrical stimulation on rat soleus muscle was demonstrated *in vitro* by MacPherson et al. (90). Further research from Ramos et al. demonstrated a significant increase in PLIN5 levels in rat skeletal muscle mitochondria following acute electrical stimulation-induced contraction; this increase was concurrent with enhanced FA oxidation capacity (90). In summary, these findings indicate acute exercise initiates the rapid translocation of PLIN5 from LDs to mitochondria (or to LD-mitochondria contact sites). This process facilitates the efficient transport and oxidation of fatty acids from LDs to mitochondria.

By contrast, determining the effect of chronic exercise on total PLIN5 protein levels yields contentious and context-specific results. Total PLIN5 protein levels in skeletal muscle of healthy or pathological populations are not significantly modulated by long-term exercise training, according to several studies. This suggests differential modulation of PLIN5 expression by different exercise modalities. HIIT acts as a more potent stimulus, and its effects appear more significant in specific groups such as individuals with obesity.

Moreover, exercise’s influence on PLIN5 extends to its regulation of organelle interactions. For instance, evidence demonstrates aerobic exercise promotes MFN2 protein accumulation at liver LD-mitochondria MCSs. This enhances hepatic FA oxidation and ameliorates NAFLD (91). While this observation was made in liver tissue, it provides a strong rationale to hypothesize that exercise may similarly modulate lipid metabolism in skeletal muscle by regulating the abundance of critical MCS proteins. An interaction between MFN2 and a C-terminal truncation mutant of PLIN5 (PLIN5 CΔ) was reported by Miner et al. (92). Boutant et al. previously demonstrated that MFN2 facilitates LD-mitochondria interactions in brown adipose tissue during adrenergic stimulation by binding to PLIN1 (93). Considering that PLIN5 shares a similar N-terminal domain structure with PLIN1, it is plausible that PLIN5 regulates lipid metabolism in skeletal muscle through a similar interaction with MFN2. Confirmation of a direct PLIN5-MFN2 interaction and explanation of its functional implications, however, necessitate further experimental validation. MFN2 fulfills multiple roles concerning organelle interactions and metabolic regulation. Its functions are essential not only for outer mitochondrial membrane fusion (which regulates mitochondrial morphology, dynamics, and respiratory function) (94, 95), but also critical for maintaining the structural and functional integrity of ER-mitochondria contact sites, known as mitochondria-associated membranes (MAMs). In the MAM region, complex interaction networks involving proteins such as phosphofurin acidic cluster sorting protein 2 (PACS-2), VAMP associated Protein B And C (VAPB), and Protein tyrosine phosphatase-interacting protein 51 (PTPIP51) are established by MFN2 through its multifunctional domains; these networks contribute to regulating lipid synthesis and transport (33, 96–101). Reporting by Borquez et al. indicated that exercise reduces high-fat diet-induced mitochondrial dysfunction and ER stress, partly by increasing the number of skeletal muscle MAMs; MFN2 is hypothesized to significantly affect this mitigation (91, 102). Disruption of lipid transport in the MAM region results from MFN2 dysfunction. This disruption leads to mitochondrial dysfunction and cellular metabolic dysregulation and maintains close associations with the pathophysiology of various metabolic diseases, neurodegenerative disorders, and cancer (103). Assessing whether MFN2 interacts with PLIN5 and how this interaction might coordinately regulate LD-mitochondria and LD-MAM interactions alongside lipid metabolism in skeletal muscle under exercise or diverse metabolic states stands as a critical research priority. Moreover, some specific molecular mechanisms mediating PLIN5-dependent LD-mitochondrial contacts have been elucidated. Work by Ouyang et al. demonstrated that mitochondria-localized ras-related protein in brain 8a (Rab8a) functions as a LD receptor in skeletal muscle, binding to PLIN5 present on the LD surface. Lipid transfer from LDs to mitochondria is facilitated by this interaction, thereby enhancing β-oxidation. Activation of AMPK leads to increased levels of guanosine triphosphate (GTP)-bound Rab8a. This increase strengthens the Rab8a-PLIN5 interaction and recruits ATGL to the LD surface, promoting lipolysis (104, 105). Separately, Miner et al. demonstrated PLIN5 binding to the fatty acid transport protein 4 (FATP4) located on the outer mitochondrial membrane in C2C12 cells, mediated through PLIN5’s C-terminal domain. This interaction facilitates close apposition between LDs and mitochondria. Efficient channeling of LD-derived FAs directly to mitochondria for β-oxidation is achieved through this PLIN5-FATP4-mediated MCS, which prevents the cytosolic accumulation of free FAs and their potential toxicity. The interaction between PLIN5 and FATP4 is strengthened by PLIN5 phosphorylation, an effect that enhances fatty acid oxidation in skeletal muscle cells experiencing starvation conditions (92). In summary, while PLIN proteins are integral to lipid metabolism, their precise regulatory roles, particularly in response to exercise, are complex and multifaceted. The conflicting outcomes of PLIN5 modulation, the challenge to the canonical PKA-phosphorylation model in muscle, and inconsistent reports on its expression following chronic training underscore this complexity. These discrepancies likely arise from the interplay of exercise modality, muscle fiber composition, and the subjects’ metabolic phenotype. Collectively, these findings suggest that PLIN-mediated lipid dynamics are governed by a highly context-dependent regulatory network, warranting further investigation to resolve these inconsistencies.

### 3.4 Motion-regulated PLINs-mediated LD-lysosome interactions and lipophagy

Cells can mobilize lipids stored in LDs through the lipophagy pathway, an alternative to the canonical cytosolic lipolysis route. Lipophagy represents a specialized form of autophagy where LDs themselves are targeted as substrates. They are sequestered utilizing autophagic mechanisms and delivered to lysosomes for degradation. Lipophagy classification into macrolipophagy, microlipophagy, and CMA depends on the specific mode of substrate entry into lysosomes. Macrolipophagy involves the formation of autophagosomes engulfing LDs; these autophagosomes later fuse with lysosomes. Inside the lysosome, lysosomal acid lipase (LAL) hydrolyzes TGs into FFAs. These FFAs are then released back into the cytosol, available for mitochondrial β-oxidation or other metabolic processes. CMA presents a contrast as a highly selective autophagic pathway. It depends on cytosolic chaperone proteins, such as heat shock cognate protein 70 (Hsc70), which recognize distinct KFERQ-like motifs on substrate proteins. These chaperones mediate the direct translocation of the substrate proteins into the lysosomal lumen for eventual degradation.

CMA holds a critical role in maintaining protein homeostasis, degrading damaged proteins, and mounting responses to nutritional stress; Besides, its role in regulating lipid homeostasis has gained recent recognition. Specific members of the PLIN protein family, including PLIN2, PLIN3, and PLIN5, contain KFERQ-like motifs essential for recognition by the CMA pathway. They are therefore considered key CMA substrates (47, 106). Chaperone proteins such as Hsc70 perform recognition and binding of PLINs. This action facilitates the selective delivery of PLINs to lysosomes where degradation occurs. PLINs are acknowledged as gatekeepers coating the LD surface; they restrict access by lipases and other factors to the neutral lipid core. For this role, PLIN degradation is viewed as a critical prerequisite or regulatory step needed to initiate LD breakdown, regardless of whether breakdown occurs through cytosolic lipolysis or lipophagy. Steric hindrance is reduced by PLIN degradation. This reduction allows lipolytic enzymes (e.g., ATGL) and the autophagy machinery better access to LDs, thereby promoting lipid mobilization. PLIN2 protein is essential for maintaining the stability of LDs, and its overexpression has the potential to cause pronounced myocardial steatosis (107). Therefore, accurate regulation concerning PLIN2 degradation is crucial to prevent excessive accumulation of lipids. Initial identification of PLIN2 as a CMA substrate was derived from research by Kaushik et al. Their findings indicated that Hsc70 binding to PLIN2 promotes PLIN2 phosphorylation by AMPK, an event facilitating lysosomal recognition and degradation (107). Reports also identify PLIN3 and PLIN5 as CMA substrates. Impaired PLIN degradation can occur due to impaired CMA function, potentially causing excessive PLIN accumulation on the LD surface. Such accumulation could, accordingly, inhibit both lipolysis and lipophagy activity, finally leading to intracellular lipid accumulation. This process is associated with lipid metabolism disorders, particularly non-alcoholic fatty liver disease (NAFLD) (47). However, the role of PLIN2 in lipophagy demonstrates complexity. Studies conducted by Mardani et al. unexpectedly indicated increased triglyceride accumulation in cardiomyocytes from PLIN2 knockout mice; this was accompanied by reduced co-localization between LDs and lysosomes (107). The notion that PLIN2 degradation promotes lipid mobilization is challenged by this observation. A plausible explanation, especially considering the role of PLIN2 in β-cells (section 3.2), suggests PLIN2 might be more than a mere CMA substrate whose degradation initiates lipolysis or lipophagy. It could potentially be directly involved in mediating LD-lysosome interactions or in regulating the lipophagy process itself. In the absence of PLIN2, while inhibition of certain lipolytic enzymes might be lessened, lipid shunting through alternative routes (e.g., dysregulated flux to mitochondria) could occur if PLIN2-mediated trafficking to lysosomes or the following FA processing pathways are simultaneously blocked. This scenario could finally result in net lipid accumulation or reduced clearance capacity. These possibilities emphasize the complex nature of PLIN2 acting as a key regulator in LD metabolism; whereas, research concerning PLIN5 regulation through the lipophagy pathway in tissues such as skeletal muscle is scarce. Moreover, whether PLIN5 itself participates in mediating interactions between LDs and lysosomes remains largely unevaluated and requires future study.

Research on how exercise mediates effects on lipophagy through LD -lysosome interactions is sparse; the majority of existing evidence can be derived from studies on liver, adipose, and cardiac tissues. Lipophagy in these specific tissues controls lipid turnover when there is an excess of nutrients or an increase in energy requirements (108–111). PLINs (especially PLIN2 and PLIN3) function as CMA substrates; therefore, exploring whether exercise can modulate lipophagy in skeletal muscle by means of CMA-mediated PLIN degradation is a necessary area of study. Studies have demonstrated that prolonged exercise activates and phosphorylates AMPK, leading to downregulation of PLIN2, a LD-associated protein, in murine liver tissue, while concurrently upregulating the expression and activity of lysosomal acid lipase (LIPA). Suppression of PLIN2 or overexpression of LIPA reduces p62 expression and enhances LC3 autophagosome formation, indicating that the AMPK-PLIN2-LIPA axis may mediate lipophagy as a critical mechanism underlying exercise-induced amelioration of hepatic lipid accumulation. Although this research primarily investigates liver tissue, it offers valuable insights into the mechanisms by which exercise facilitates LDs degradation in skeletal muscle (112). Although this research focuses on hepatic tissue, it provides valuable insights into the mechanisms by which exercise facilitates LD degradation in skeletal muscle. The findings indicate that exercise may enhance the chaperone-mediated autophagy (CMA)-dependent degradation of perilipins (PLINs) through activation of the AMPK-LIPA signaling pathway or modulation of molecular components such as Hsc70 and LAMP2A, thereby alleviating the suppression of LD catabolism.

**Figure 1:**
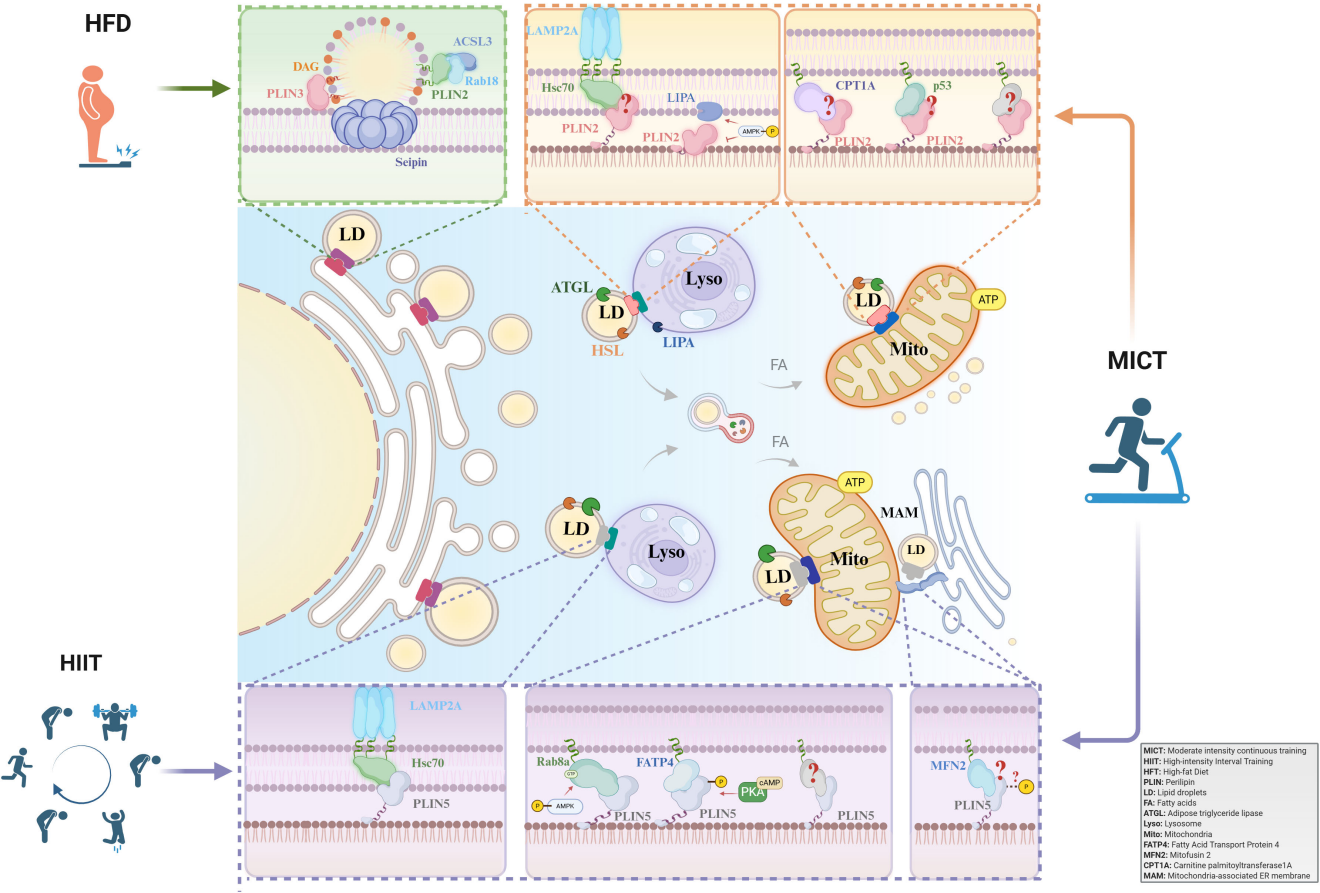
The role of PLIN-mediated lipid droplet-organelle interactions in exercise adaptation. PLINs are key regulators of LDs in skeletal muscle. While PLIN3 governs LD biogenesis, PLIN2 and PLIN5 mediate lipolysis in response to exercise. Acute exercise depletes PLIN2/5-coated LDs. In contrast, long-term training elicits adaptations: MICT upregulates PLIN2 and PLIN5, whereas HIIT specifically enhances PLIN5, promoting LD-mitochondria coupling and improving fatty acid oxidation. MICT, Moderate-intensity continuous training; HIIT, High-intensity interval training; HFT, Hight-fat Diet; PLIN, Perilipin; LD, Lipid droplet; FA, Fatty acid; ATGL, Adipose triglyceride lipase; Lyso, Lysosome; Mito, Mitochondria; FATP4, Fatty Acid Transport Protein 4; MFN2, Mitofusin 2; CPT1A, Carnitine palmitoyltransferase 1A; MAM, Mitochondria-associated membranes.

In summary, sustained physical exercise, particularly endurance and high-intensity interval training, profoundly modulates the expression and subcellular localization of Perilipin (PLIN) proteins in skeletal muscle. Members of the PLIN family are pivotal in mediating the exercise-induced mobilization of intramyocellular triglycerides, thereby augmenting fatty acid oxidation and fostering long-term metabolic adaptations, as illustrated in **Figure 1**. This exercise-centric regulatory network also underscores the potential of PLINs as pharmacological targets for metabolic disorders. Notably, key signaling pathways activated by exercise, such as PPAR, AMPK, and PKA signaling, are themselves druggable. For instance, agents including fibrates acting via PPARs, metformin via AMPK, and adrenoceptor modulators via PKA signaling have been demonstrated to regulate the expression and activity of PLIN2 and PLIN5. This convergence suggests that pharmacological interventions could mimic or potentiate the beneficial effects of exercise on PLIN-mediated lipid metabolism. Nevertheless, further investigation is required to elucidate the precise mechanisms by which different exercise modalities modulate the functions of various PLIN isoforms and their downstream effects. A more in-depth exploration of these molecular pathways is essential for establishing a theoretical foundation upon which optimized exercise regimens and targeted therapeutic strategies for preventing and treating metabolic diseases can be developed.

## 4 Conclusion and future perspectives

PLINs play a critical role in regulating skeletal muscle lipid homeostasis. Specific exercise modalities exert a profound regulatory influence on skeletal muscle lipid metabolism, primarily by modulating the expression of key regulatory proteins within the PLINs on the surfaces of LDs. These proteins orchestrate dynamic interactions between LDs and intracellular organelles, such as the endoplasmic reticulum, mitochondria, and lysosomes, thereby facilitating efficient transport of fatty acids to mitochondria for β-oxidation or degradation via lipophagy. This process mitigates aberrant intramuscular triglyceride accumulation and the generation of deleterious lipid intermediates. Long-term, structured exercise regimens, particularly MICT and HIIT, induce adaptive changes in skeletal muscle PLINs protein levels more effectively than AE bouts. MICT upregulates PLIN2 and PLIN5 expression, promoting intramuscular triglyceride hydrolysis and FAO while stabilizing LD-mitochondrial contacts. HIIT, as a more potent stimulus, particularly in populations such as obese individuals, markedly enhances PLIN5 expression, augments mitochondrial quality and function, and ameliorates ectopic lipid deposition, thereby bolstering FAO capacity. Although acute exercise elicits minimal changes in total PLIN protein levels, it selectively depletes LDs coated with PLIN2 and PLIN5. It promotes rapid PLIN5 translocation to LD-mitochondrial contact sites, enhancing fatty acid transport and oxidation. However, a complete understanding is still lacking regarding the exact mechanisms through which PLINs direct the dynamic interactions between LDs and other organelles such as the endoplasmic reticulum, mitochondria, and lysosomes. Additional molecular analysis is also necessary to determine the specific effects of various exercise modalities (e.g., endurance exercise and high-intensity interval training) on these inter-organellar interactions and to clarify their contribution to promoting efficient lipid utilization or storage.

To create a more detailed and accurate molecular map of how exercise regulates skeletal muscle lipid metabolism, future research efforts need to prioritize an in-depth investigation into the mechanisms governing PLINs-mediated organelle dynamics and their responsiveness to exercise stimuli. A key priority is to identify the specific post-translational modifications on PLIN5 that serve as molecular switches for LD-mitochondria tethering, and to determine how these are differentially engaged by acute HIIT versus MICT. Such discoveries would illuminate the rapid signaling cascades that fine-tune lipid utilization in response to immediate metabolic demands. Furthermore, exploring the role of PLIN2 ubiquitination in exercise-induced lipophagy is a compelling avenue. Establishing this link would uncover a novel, alternative pathway for LD turnover and cellular quality control during recovery from strenuous activity. Finally, a more nuanced understanding of the functional interplay between PLIN2 and PLIN5 in chronically trained muscle is essential. Investigating whether these proteins demarcate distinct LD subpopulations destined for either immediate oxidation or transient storage will refine our models of intramuscular lipid trafficking.

In conclusion, this study establishes a sophisticated network model of exercise-induced PLINs-organelle interactions, elucidating the multifaceted pathways through which exercise modulates skeletal muscle lipid homeostasis. The model offers innovative perspectives and prospective molecular targets for devising exercise-based therapeutic strategies targeting PLINs, thereby facilitating more efficacious and precise interventions for the prevention and management of metabolic disorders, including obesity and diabetes. Such advancements are expected to offer novel mechanistic understandings and identify new therapeutic targets, and accordingly, facilitate the development of PLIN-targeted exercise interventions to produce more effective and accurate approaches for preventing and treating metabolic conditions such as obesity and diabetes.

## Glossary

FA: Fatty acid
TG: Triglyceride
IMTG: Intramuscular triglyceride
LD: Lipid droplet
LRP: Lipid droplet-associated protein
PLIN: Perilipin
MCS: Membrane contact site
ATGL: Adipose triglyceride lipase
HSL: Hormone-sensitive lipase
CPT1A: Carnitine palmitoyltransferase 1A
DAG: Diacylglycerol
CMA: Chaperone-mediated autophagy
Hsc70: Heat shock protein 70
PPARs: Peroxisome proliferator-activated receptors
CD36: Cluster of differentiation 36
LPL: Lipoprotein lipase
CGI-58: Comparative gene identification-58
PKA: Protein kinase A
LAMP2A: Lysosomal-associated membrane protein 2A
FABP: Fatty Acid-Binding Protein
FATP4: Fatty Acid Transport Protein 4
LTP: Lipid transfer protein
DGAT: Diacylglycerol acyltransferase
AH: Amphipathic helix
ACSL3: Acyl-CoA long-chain synthetase 3
LPS: Lipopolysaccharides
PFKL: Phosphofructokinase liver
AFB1: Aflatoxin B1
GSIS: Glucose-stimulated insulin secretion
MAM: Mitochondria-associated membranes
PDM: Peridroplet mitochondria
CM: Cytoplasmic mitochondria
AMPK: AMP-activated protein kinase
MFN2: Mitofusin 2
PTPIP51: Protein tyrosine phosphatase-interacting protein 51
VAPB: VAMP associated Protein B And C
PACS-2: Phosphofurin acidic cluster sorting protein 2
LAL: Lysosomal acid lipase
NAFLD: Non-alcoholic fatty liver disease
T2DM: Type 2 diabetes mellitus
AE: Aerobic exercise
FAO: Fatty acid oxidation
HIIT: High-intensity interval training
MICT: Moderate-intensity continuous training
EPOC: Excess post-exercise oxygen consumption
LIPA: Lysosomal acid lipase
ACAT1: Acetyl-Coenzyme A acetyltransferase 1
FGF21: Fibroblast growth factor 21
p-PLIN5: Phosphorylated PLIN5
PTM: Post-translational modification
CaMK: Calcium/calmodulin-dependent protein kinases
VO_2max_: Maximal oxygen consumption
Pex30: Peroxin 30
Rab8a: Ras-related protein in brain 8a
GTP: Guanosine triphosphate.
